# Functional specialization of the left ventral parietal cortex in working memory

**DOI:** 10.3389/fnhum.2014.00440

**Published:** 2014-06-18

**Authors:** Jennifer Langel, Jonathan Hakun, David C. Zhu, Susan M. Ravizza

**Affiliations:** ^1^Neuroscience Program, Michigan State UniversityEast Lansing, MI, USA; ^2^Department of Psychology, Michigan State UniversityEast Lansing, MI, USA; ^3^Department of Radiology, Michigan State UniversityEast Lansing, MI, USA

**Keywords:** stimulus-driven attention, voluntary attention, short-term memory, working memory, verbal working memory, language

## Abstract

The function of the ventral parietal cortex (VPC) is subject to much debate. Many studies suggest a lateralization of function in the VPC, with the left hemisphere facilitating verbal working memory and the right subserving stimulus-driven attention. However, many attentional tasks elicit activity in the VPC bilaterally. To elucidate the potential divides across the VPC in function, we assessed the pattern of activity in the VPC bilaterally across two tasks that require different demands, an oddball attentional task with low working memory demands and a working memory task. An anterior region of the VPC was bilaterally active during novel targets in the oddball task and during retrieval in WM, while more posterior regions of the VPC displayed dissociable functions in the left and right hemisphere, with the left being active during the encoding and retrieval of WM, but not during the oddball task and the right showing the reverse pattern. These results suggest that bilateral regions of the anterior VPC subserve non-mnemonic processes, such as stimulus-driven attention during WM retrieval and oddball detection. The left posterior VPC may be important for speech-related processing important for both working memory and perception, while the right hemisphere is more lateralized for attention.

## INTRODUCTION

The ventral parietal cortex (VPC) is a large region encompassing both the supramarginal and angular gyri, and its function has been the subject of much study and debate in cognitive neuroscience (Cabeza et al., 2012; Mars et al., 2012; Carter and Huettel, 2013). In particular, the temporal parietal junction (TPJ), a region residing at the intersection of the superior temporal sulcus, inferior parietal cortex, and occipital cortex (Mars et al., 2012), has been ascribed a wide range of functions ranging from attention (Corbetta et al., 2000; Todd et al., 2005; Serences and Yantis, 2007; Weissman and Prado, 2012; Chang et al., 2013; Chica et al., 2013), auditory-motor integration (Hickok and Poeppel, 2000), updating contextual cues (DiQuattro and Geng, 2011; DiQuattro et al., 2013), binding of episode features (Elman et al., 2013) and social cognition (Saxe and Kanwisher, 2003). Anterior regions of the supramarginal gyrus, outside the TPJ, have also been attributed a wide variety of functions including verbal working memory storage (Paulesu et al., 1993; Jonides et al., 1998) and sensory motor processing (Schwartz et al., 2012; Warbrick et al., 2013). Some of these functions are claimed to be lateralized; for example, stimulus-driven attention and social cognition are claimed to be lateralized to the right hemisphere whereas speech and language processing (e.g., verbal working memory storage, auditory-motor integration) are more dominant in the left hemisphere (Duncan et al., 1999; Szczepanski et al., 2010; Szczepanski and Kastner, 2013).

Determining the functions of the VPC and whether they are heterogeneous or homogenous has been a frequent subject of research (Cabeza et al., 2008, 2012; Mars et al., 2012; Nelson et al., 2012; Carter and Huettel, 2013). Some have argued that the VPC has a unitary function (Cabeza et al., 2012; Carter and Huettel, 2013) while others have argued that functions across the VPC are most likely “fractionated” (Mars et al., 2012; Nelson et al., 2012). The goal of the present study was to assess differences in the pattern of VPC recruitment in two domains in order to observe the overlap in functional activity. Knowing the pattern of activity of this region across tasks and how activity relates to performance will provide a basis for future hypotheses concerning the common or dissociable functions of the VPC.

Much research has focused on how the right VPC might contribute a common process across many cognitive domains (Corbetta et al., 2008; Cabeza et al., 2012; Carter and Huettel, 2013), however, we were especially interested in functions of the left hemisphere. Similar to the right VPC, the left VPC has been implicated in many different tasks; that is, the left VPC is argued to be important for working memory processes involved in storing verbal information (Paulesu et al., 1993; Salmon et al., 1996; Gruber and von Cramon, 2001; Barch and Csernansky, 2007) as well as non-mnemonic processes such as updating sensory and motor context (Geng and Vossel, 2013). Of course, there may be a common process that the left VPC contributes to these tasks, but as a first step, we examined whether such tasks evoked activity in common or dissociable regions in the left VPC. Toward this end, we examined the pattern of activity in two different tasks that reliably evoke activity in the left VPC – verbal working memory and oddball detection – that differ in the demand they place on the memory system.

The left VPC has been associated with verbal working memory functions based on lesion and imaging data. For example, lesions associated with selective verbal WM deficits are typically located in the left temporoparietal cortex (Shallice and Vallar, 1990) with the greatest degree of overlap in the left VPC (Graves et al., 2005). Left VPC activity is also observed in neuroimaging studies of verbal WM when contrasted with non-verbal WM (Paulesu et al., 1993; Salmon et al., 1996; Gruber and von Cramon, 2001; Barch and Csernansky, 2007). Some have proposed that this region acts as a dedicated storage buffer as proposed by Baddeley and Hitch (1974) and Baddeley (2000), whereas others have suggested that this region integrates phonological and motor codes that facilitates verbal WM encoding and maintenance (Hickok and Poeppel, 2000; Hickok et al., 2009).

Detection of oddball or novel stimuli reliably evokes bilateral activity in the VPC. Oddball tasks are argued to recruit the VPC because infrequent events capture attention (Corbetta et al., 2008). Accordingly, other studies such as those examining contingent capture from task-relevant stimuli have also reported bilateral VPC activity (Serences et al., 2005). These findings and others have been the basis for the proposal that this region is involved in stimulus-driven attention (Cabeza et al., 2012), although other non-mnemonic functions such as sensorimotor integration (Carter and Huettel, 2013) or context updating (DiQuattro et al., 2013) have also been proposed. Importantly, there is little support for a strict lateralization of these functions in the VPC. For example, many studies of oddball detection find bilateral responses in the VPC to rare targets (c.f., Kiehl et al., 2001, 2005; Laurens et al., 2005; Wolf et al., 2008). Moreover, an increasing number of studies have found engagement of the left VPC during stimulus-driven capture (Wolf et al., 2008; Weidner et al., 2009; Doricchi et al., 2010).

We chose oddball detection and verbal working memory tasks because they evoke reliable activity in the left VPC, but differ in the demands they place on the memory system. In our oddball detection task, participants have to respond to any stimulus that is different than the standard stimulus. They do not have to remember a pre-specified target and, thus, working memory demands are relatively low. We also examined activity in each stage of a verbal working memory task – encoding, maintenance, and retrieval. Encoding and oddball detection are similar in that they both require attention and visual perception whereas maintenance and oddball detection are quite different in terms of processing internal or external stimuli. Examining the pattern of overlap in these two tasks can provide some insight into VPC function in the left hemisphere. For example, left VPC activity at encoding in WM and during oddball detection might suggest a role of this region in visual processing or attention, but not in WM storage.

In a previous study of verbal WM (Ravizza et al., 2011), we found that anterior portions of the VPC were active during encoding and retrieval, but not during maintenance. In contrast, the posterior segment of the VPC in the temporal–parietal junction was active during all working memory stages. In that paper, we suggested that the posterior VPC was important for storing verbal information because of its involvement in speech perception. The anterior VPC was proposed to have a non-mnemonic contribution to verbal working memory performance perhaps via stimulus-driven attention. Thus, we predict that the left anterior VPC will be active in both encoding and retrieval stages of the WM task and in the oddball detection task. For the left posterior VPC, we predict that it will be active in the WM task, but not during oddball detection. Although we are focused on the left VPC, patterns of activity will also be reported for the right hemisphere in order to compare potential differences in laterality.

## MATERIALS AND METHODS

### PARTICIPANTS

Twenty right-handed individuals (11 F/9 M) between the ages of 20 and 29 (average age ± SD, 21.5 ± 2.1) participated in this study. All participants were paid US $20 for their participation and provided informed consent following the procedures approved by the Human Research Protection Program at Michigan State University. Data from three participants were excluded from all analyses. Participants were excluded either because of technical difficulty with the scanner, a failure to follow task instructions, or chance performance in the object WM task. Thus, data from 17 participants were analyzed. Due to a technical difficulty, behavioral data for one participant was unreliable for the oddball task. This participant was included in all other analyses.

### STIMULI

Seventeen English consonants presented in 36-point Arial font composed the set of verbal items. The set of object stimuli consisted of seventeen Korean letters of similar size and complexity to the English letters (Paulesu et al., 1993). All subjects confirmed that they did not know the Korean language. The stimuli were centered on a 32′′ LCD monitor with a 1024 × 768 matrix resolution and subtended.88° and 1.3° of visual angle.

### EXPERIMENTAL DESIGN

**FIGURE 1:**
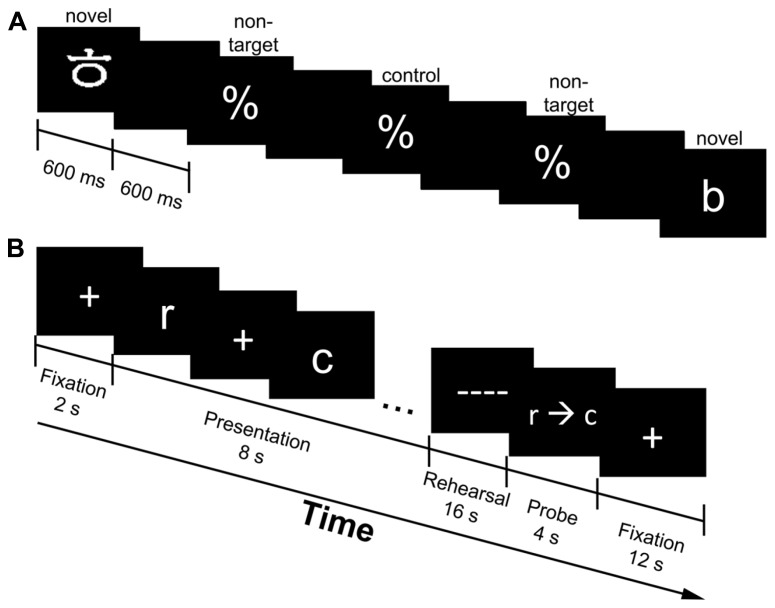
**Experimental design of the oddball **(A)** and working memory [WM; **(B)**] task.** The oddball task consisted of novel targets (Korean and English letters), non-targets (percentage signs) and control targets (same as non-targets, but used as the baseline condition for comparison purposes; one-third of the time the percentage signs were denoted as the control targets); stimulus duration was 600 ms, while the interstimulus interval was 600 ms. **(A)**. Presented is an example sequence of a novel target (Korean letter) followed by a series of standard stimuli (%) and another novel target (English letter). Participants indicated whenever they saw a stimulus that was not a percentage sign. The WM task consisted of verbal (English letters only) and object (Korean letters only) trials. Presented is a verbal trial followed by a correctly ordered probe **(B)**. A fixation cross marked the onset of each trial (2 s), followed by the presentation of five randomly selected stimuli (all verbal or all object; 8 s). Participants were to rehearse the stimuli in serial order (16 s) and make a response when the probe (r→c) was presented (4 s). A fixation cross marked the end of the trial (12 s).

Behavioral data were collected via the E-prime software package (Psychology Software Tools) with a fiber optic response keypad. For the oddball task, participants were shown a series of percent signs (%) interspersed with novel target stimuli at random times (**Figure 1A**). Novel targets consisted of English and Korean letters and occurred with a minimum of 14.4 s and a maximum of 25.2 s from the time of the last target. The jitter in the oddball task was set in increments of 1800 ms; specifically, oddball events could occur at one of seven times (14.4, 16.2, 18, 19.8, 21.6, 23.4, or 25.2 s) after the previous oddball target. Participants were asked to press their right index finger on the keypad whenever they saw a stimulus that was not a percentage sign. Stimulus duration was 600 ms and a blank screen was presented for 600 ms between stimuli. A subset of the standard stimuli (percent signs) were used as the baseline condition, so that we could isolate activity to the standard stimulus from that associated with novel targets; this was denoted as the “control target” for the task. These control targets were randomly selected percent signs with the same presentation constraints as the novel targets. The oddball task was completed in five runs (4 min 48 s each) and the total number of trials per condition varied from 15 to 30, since the novel targets were presented at random times. On average, 49.3% of the novel targets were object stimuli, while 50.7% were verbal stimuli.

The WM task consisted of five runs of eight trials each [four verbal (English letter trials) and four object (Korean letters trials) in random order] and total trial duration was 42 s (5 min 44 s/run). The sequence of each trial was as follows (refer to **Figure 1B**). A fixation cross appeared at the onset of each trial for 2 s. During the encoding phase, a sequence of five randomly selected stimuli (all verbal or all object) was displayed across an 8 s interval. Each stimulus in the sequence was displayed for 1 s with an induced temporal jitter between 200 and 1000 ms between stimuli. The jittering scheme resulted in the constant length of 8 s for the encoding interval. Participants were instructed to rehearse the stimuli in serial order during a maintenance period (16 s), which was indicated by a series of dashed lines (––) that appeared on the screen. A probe (r→c) initiated the start of the retrieval period and participants were asked to indicate whether the stimuli were in the order in which they were presented. The probe was in the correct order half of the time, while two adjacent letters from the list were swapped the other half of the time. Participants indicated a correct sequence by pressing their right index finger, while incorrect sequences were indicated by pressing their right middle finger on the response box. Participants had 4 s to make a response before a fixation cross appeared on the screen for 12 s marking the end of the trial. Only subsets of fMRI time points were analyzed for each stage of the WM task in order to reduce the overlap of activity (see *ROI analysis* for more detail).

The WM and oddball tasks were completed on the same day, but all WM runs were completed before the oddball runs. To ensure that WM demands were low in the oddball task, runs of the WM task were not alternated with runs of the oddball task so that participants were less likely to memorize oddball targets. The WM task was completed first, since it is more cognitively demanding than the oddball task. All participants practiced the two tasks briefly before entering the scanner to ensure that they understood the task directions.

### fMRI ACQUISITION

Imaging was performed on a GE 3 T Signa HDx scanner with high-order shimming applied for improvement of local field homogeneity. Functional images were collected using gradient echo planar imaging [TR = 1.8 s (oddball task), 2 s (WM task); echo time = 28 ms; voxel size = 3.44 mm × 3.44 mm × 3.8 mm; flip angle = 74° (oddball task) 77° (WM task); field of view = 220 mm). TRs differed between the WM and oddball detection tasks because of the faster timing of the oddball detection task. Twenty-eight axial slices were collected for both tasks in an interleaved fashion with a total of 156 scans/run in the oddball task and 168 scans/run in the WM task. Sagittal high resolution T_1_-weighted structural scans (voxel size = 1.5 mm × 0.938 mm × 1.25 mm) were also acquired from each participant for anatomical registration.

### PREPROCESSING

Preprocessing and first-level analysis of the functional MRI data was performed using FEAT (v5.98) within FSL (FMRIB’s Software Library; Smith et al., 2004). Preprocessing consisted of motion correction through MCFLIRT (Jenkinson et al., 2002), brain extraction through the FSL brain extraction tool (BET; Smith, 2002), spatial smoothing with a Gaussian kernel of full width at half maximum (FWHM) of 9 mm, and high-pass temporal filtering (<0.01 Hz). Each participant’s functional MRI scans were registered to their own high resolution T1 structural scan (linear transformation; *df *= 7) followed by registration to a standard space image (MNI 152; linear transformation; *df* = 12). FILM (FMRIB’s Improved Linear Model) was used on the single-subject data. This method uses auto-correlation correction to pre-whiten each voxel’s time series, which provides better efficiency of the model (Woolrich et al., 2001).

### WHOLE-BRAIN ANALYSIS

Higher-level group analysis was done using FLAME (FMRIB’s Local Analysis of Mixed Effects). FLAME uses each participant’s time series data to model the participant’s activity in each condition. Individual time course vectors describing the onset of each event were convolved with a canonical double-gamma hemodynamic response function (HRF), along with its temporal derivative, and entered as predictors in a modified general linear model (GLM). In the oddball task, four regressors were included in the final model: correct (detected) targets, incorrect targets, control targets and non-targets (standard stimulus; refer to **Figure 1A**). Due to the fact that there were no differences found between verbal and object targets in the oddball task (no clusters found at *Z* = 3.1, *p* = 0.001) these conditions were combined in the final model. To identify regions responsive to targets during the oddball task, a two-sample paired *t*-test was conducted between correct novel and control targets. Results of this contrast were thresholded at FWE-corrected *p* < 0.05.

For the WM task twelve regressors were included in the model: 2 (verbal versus object trials) × 3 (encoding versus maintenance versus retrieval stages) × 2 (correct versus incorrect trials). One-way *t*-tests were performed to identify significant (above baseline; mean response different from zero) regions of activation at each stage of the task (correct encoding, maintenance, and retrieval). Baseline in this analysis included the unmodeled portion of the signal. All resulting images were thresholded at FWE-corrected *p* < 0.05.

### TIME COURSE ANALYSIS

Time courses for VPC ROIs produced in the whole-brain analysis were calculated using a mean time series extraction utility in FSL. We considered activity as residing in the VPC if the peak voxel was located in the supramarginal or angular gyrus of the parietal cortex below the intraparietal sulcus (*z* < 30 in Talairach space). Given potential smoothing errors, peak coordinates in the group analysis that fell within the superior temporal gyrus were also considered part of the VPC if adjacent voxels appeared in the parietal cortex.

For the oddball task, percent change in signal intensity was calculated as the difference between signal intensity at each time point relative to the start of each trial. For the WM task, we assessed percent signal change at points of minimal overlap between each stage of the serial-recognition task in which the hemodynamic response function (HRF; 4–6 s lag time) would be at its peak (see Ravizza et al., 2011). The points corresponded to 6–12 s after the onset of the trial for encoding, 20–26 s after trial onset for maintenance, and 28–34 s after trial onset for retrieval. Thus, the likelihood of overlap of activity for encoding and maintenance is very low. However, potentially there is activity overlap in the retrieval stage which occurs at the later time points. The first two and last two time points of each trial were used to assess baseline activity. Paired *t*-tests were used to compare signal intensity at each stage to baseline, as well as for comparisons between signal intensity for the verbal and object condition at each stage. For all ROI-based analyses, *t* values with *p *< 0.05, uncorrected were considered statistically significant.

The whole-brain analysis of the oddball task produced a large cluster encompassing the anterior parietal, motor, and premotor cortices in the left hemisphere. To isolate anterior VPC activity, a 5 mm radius spherical mask centered on the VPC peak in the left hemisphere was created. This mask was registered to subject space where the average time series for all voxels falling within the mask was extracted. This region of the left anterior VPC overlapped with the VPC ROIs generated by the whole-brain analysis of WM retrieval, and the mask was subsequently used when extracting time course data from the WM task as well. This ensured that we isolated activity in the anterior VPC as well as targeting only voxels that were active in both tasks.

The right aVPC sites and the left posterior VPC regions were more focal in the inferior parietal cortex and superior temporal gyrus. For these sites, time courses were extracted from the entire ROI produced in the whole-brain analyses.

## RESULTS

### BEHAVIORAL RESULTS

**FIGURE 2:**
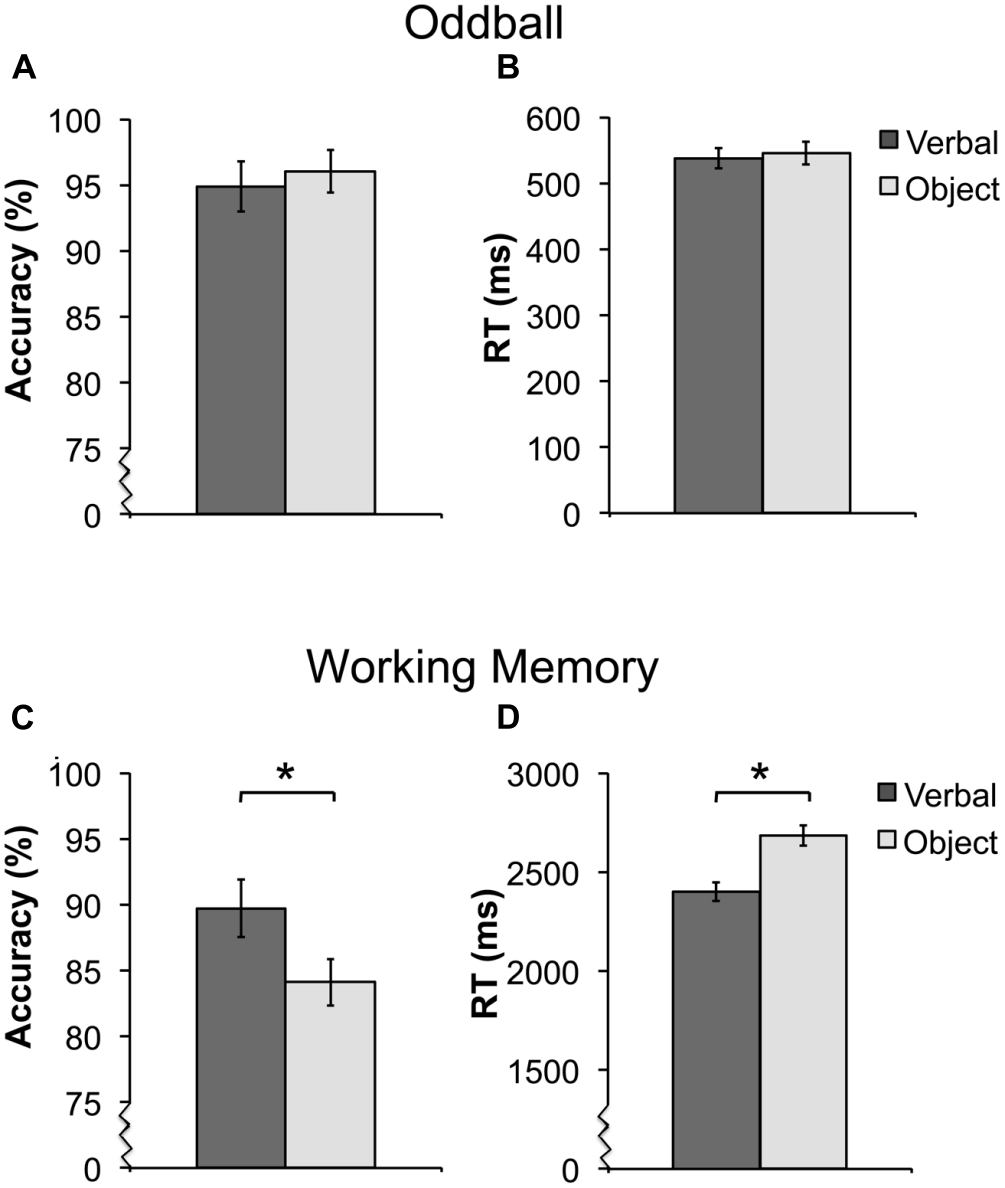
**Average accuracy and reaction time (RT) for the oddball [**(A,B)** ± SEM] and working memory [WM; **(C,D)** ± SEM] task.** For the oddball task, there were no differences in detecting verbal or object targets in accuracy **(A)** or RT **(B)**. For the WM task, participants had both higher performance **(C)** and faster RT **(D)** for verbal trials. *Asterisks indicate a significant difference between the verbal and object trials of the WM task at *p* < 0.05. For better visualization of the differences in accuracy and RT, the y-axis starts a higher value in **(A,C,D)**, this is represented by the jagged line in these graphs.

Participants were highly accurate at detecting novel targets (mean = 95.5%). There were no differences in detecting verbal or object targets in accuracy [*t*(15) = 0.92, *p* = 0.37) or RT [*t*(15) = 1.22, *p* = 0.24; **Figures 2A,B**). In the WM task, participants performed better on verbal trials compared to object trials. Accuracy was greater [*t*(16) = 2.73, *p* < 0.05], and reaction time (RT) was faster [*t*(16) = 3.7, *p* < 0.05) for the verbal condition compared to the object condition (**Figures 2C,D)**.

### ODDBALL TASK

**FIGURE 3:**
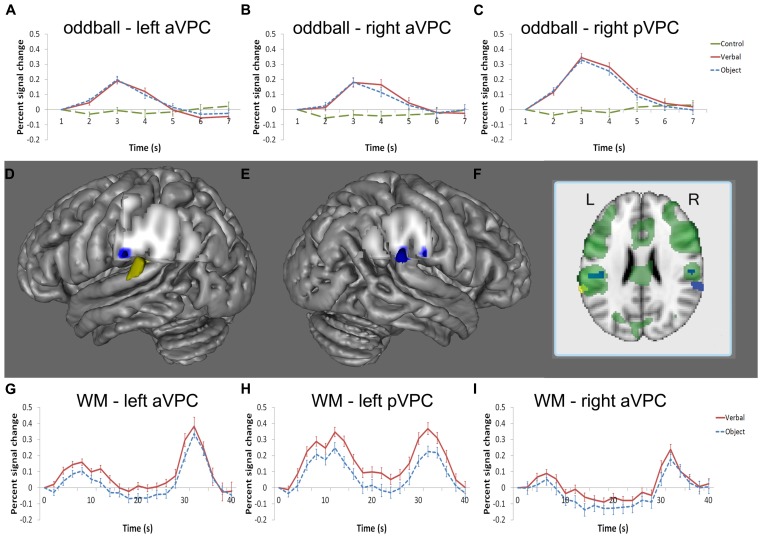
**Regions of the VPC active in the oddball and WM tasks.** Blue indicates significant activity in the oddball task, yellow indicates significant activity at encoding in the WM task, and green denotes significant activity at retrieval in the WM task **(D–F)**. Left and right aVPC regions [left: **(D)**; right: **(E)**] in the supramarginal gyrus were active in the oddball task [left: **(A)**; right: **(B)**] and at retrieval during the WM task [left: **(G)**; right: **(I)**]. A separate region in the left STG/VPC **(D)** was active at encoding and retrieval **(H)**, but not for oddball detection whereas the homologous region in the right hemisphere **(E)** displayed the opposite pattern; namely, the right STG/VPC was reliably active in the oddball task **(C)** but not the WM task. The BOLD percent signal change is defined with respect to the image signal of the first time point of a trial, which is at the baseline. Note that only activity in regions of the STG and VPC is shown in the figure.

The whole brain analysis of the oddball task was used to identify regions responsive to oddball targets. No differences were found when comparing verbal and object oddball targets in the whole brain analysis so these conditions were collapsed and compared to the baseline condition. Consistent with other studies of oddball detection (c.f., Kiehl et al., 2001, 2005; Laurens et al., 2005; Wolf et al., 2008), the group contrast between correct target > control target in the oddball task revealed activation across a large area of the parietal cortex, including the left and right VPC (**Figure 3**). The peak of activation in the left VPC resided in the supramarginal gyrus (Talairach coordinates: -52, -25, 24; **Figure 3A**). Two regions of the right VPC were also sensitive to oddball targets in the whole-brain analysis. One region was homologous to the left VPC region in the right anterior VPC (aVPC; Talairach coordinates: 52, -24, 24; *k* = 26; described above (**Figure 3B**) and the other region had peak activity more posterior in the VPC (pVPC; Talairach coordinates: 59, -37, 24; *k* = 141) and included part of the superior temporal gyrus (**Figure 3C**).

### WM TASK

We also measured activity in different stages of the verbal WM task to assess whether VPC regions engaged in WM differed from those observed in the oddball task. Significant activity in the left VPC was observed during encoding in the whole-brain analysis (Talairach coordinates: -62, -40, 20). This more posterior region in the VPC included part of the supramarginal and superior temporal gyrus and did not overlap with the left aVPC region found in the oddball task (**Figures 3F,H**). In *post hoc* analyses, this left pVPC/STG region tended to be more active for verbal information than objects, but the comparison did not reach significance [*t*(16) = 2.02, *p* = 0.061]. Other regions found to be active during encoding, maintenance, and retrieval for verbal versus baseline are displayed in **Table 1**.

**Table 1:** Global *Z*-max values and Talairach coordinates for areas active when comparing verbal versus baseline for encoding, maintenance, and retrieval.

Structure	Hem.	*X*	*Y*	*Z*	BA	*Z*-max	*k*
**Encoding**
Precentral gyrus	L	-47	-3	37	6	7.93	50484
^*^Frontal operculum, inferior frontal gyrus, inferior temporal gyrus, precentral gyrus, supramarginal gyrus, lateral occipital cortex, cingulate gyrus, supplementary motor cortex, cerebellum	L and R			
Superior temporal gyrus	L	-62	-40	20	22	6.2	205
Superior temporal gyrus	R	65	-36	12	22	5.54	97
Paracentral lobe	R	2	-41	60	5	4.6	29
Middle frontal gyrus	L	-25	50	-12	11	4.91	17
Temporal lobe; sub-gyral	R	37	-9	-22	20	4.65	17
**Maintenance**
Precentral gyrus	L	-49	-3	41	6	5.94	207
Medial frontal gyrus	L	-7	3	54	6	5.44	166
Parietal lobe; Insula	L	-33	-42	26	13	5.16	154
Medial frontal gyrus	R	26	32	15	9	5.1	125
Cerebellum; Culmen	R	25	-57	-26	NA	4.84	47
Precentral gyrus	L	-48	11	5	44	4.61	17
**Retrieval**
Inferior parietal lobe	L	-45	-34	46	40	7.73	73744
^*^Frontal pole, frontal orbital cortex, inferior frontal gyrus, supramarginal gyrus, angular gyrus, postcentral gyrus, cingulate gyrus, middle temporal gyrus, cerebellum	L and R						
Superior frontal gyrus	L	-27	55	-11	10	5.3	165
Limbic lobe; uncus	L	-22	5	-31	28	4.81	73
Occipital lobe; cuneus	R	4	-86	28	19	4.74	14
Middle frontal gyrus	L	-43	42	-16	11	4.58	12

* Asterisks indicated areas contained in the larger clusters found in encoding and retrieval. *Z*-max values and Talairach coordinates for areas larger than 10 voxels are illustrated. **coordinates are in Talariach space; *p* < 0.05.

During the maintenance interval, no regions of the VPC were significantly active in the whole-brain analysis. However, at retrieval, a large area of the VPC was observed that overlapped with the bilateral aVPC regions supporting oddball detection and the left pVPC site supporting verbal WM encoding (see **Figure 3F** for overlap). The aVPC sites (left and right) active in the oddball task were then used as ROI’s to extract time-course information at other stages of the WM task that were not directly produced by this analysis (encoding and maintenance of verbal WM and all stages of object WM; see ROI Analysis in Methods).

Time series reflecting the percent signal change averaged across trials were extracted for each participant’s left and right aVPC ROI in the verbal and object conditions. During the verbal condition, activity of the left aVPC was significantly above baseline using the ROI-based threshold of *p* < 0.05 at encoding [*t*(16) = 4.32, *p* = 0.001], but not during the maintenance phase [*t*(16) = 0.62, *p* = 0.54] (**Figure 3G**). During the object condition, activity of the left aVPC was significantly above baseline at encoding [*t*(16) = 2.6, *p <* 0.05] and retrieval [*t*(16) = 6.42, *p <* 0.001], but not during maintenance [*t*(16) = 1.49, *p* = 0.16].

Comparisons between the verbal and object conditions revealed that left aVPC activity was greater in the verbal WM condition at encoding, but not maintenance or retrieval [encoding: *t*(16) = 2.25, *p* < 0.05; maintenance: *t*(16) = 2.04, *p* < = 0.06; retrieval: *t*(16) = 1.03, *p* = 0.32]. This pattern replicated our previous WM findings for the left aVPC region (Ravizza et al., 2011) and demonstrates that this region responds preferentially to information when external stimuli are present at encoding and retrieval.

The corresponding aVPC in the right hemisphere that was active at retrieval showed a reduction of activity during maintenance compared to baseline [*t*(16) = 2.18, *p* < 0.05] and was not reliably active during encoding even at a much reduced threshold [*t*(16) = 0.44, *p* = 0.67] (**Figure 3I**). This region showed no preference for verbal and object conditions (*p* values > 0.1).

### CORRELATION OF VPC ACTIVITY AND WM PERFORMANCE

**FIGURE 4:**
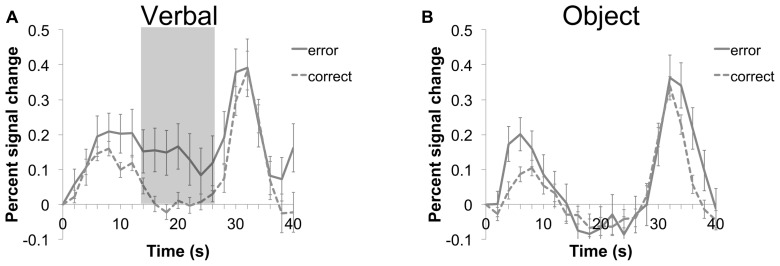
**Time course of average (±SEM) activity for the left VPC during verbal **(A)** and object **(B)** trials of the WM task.** Error (solid line) and correct (dashed line) trials are shown for each condition. A binary logistic regression indicated that higher activity averaged during the maintenance period (timepoints 14–26; shaded area) of the verbal condition was associated with a greater likelihood of making an error.

To assess whether activity of the left and right aVPC regions found in both oddball detection and WM retrieval was related to accuracy on a trial-to-trial basis, we performed a binary logistic regression using activity in the VPC as a predictor of accuracy on each trial (see **Figure 4** for average time course for error and correct trials). For this analysis, each trial (*n* = 35 errors; *n* = 305 correct items) was treated as a separate event. Within-subject factors (subject and trial) were modeled to account for correlated observations in the data set. Note that errors were not averaged across participants. For the left aVPC, higher activity at maintenance, averaged across the entire maintenance interval timepoints 14–26, was associated with a greater likelihood of making an error in memory (β = -1.09; Wald χ^2^ = 5.78, *p* < 0.05; **Figure 4**). Errors were not significantly related to activity at encoding (β = -0.7; Wald χ^2^ = 1.15, *p* = 0.28) or retrieval (β = -0.15; Wald χ^2^ = 0.18, *p* = 0.67).

Neither the aVPC site in the right hemisphere nor the pVPC site in the left hemisphere was related to performance at any stage of the WM task (R aVPC: all βs < -0.56, all (*p*-values > 0.2; L pVPC: (all βs < -0.38, all *p* values >0.29).

### HEMISPHERIC SPECIALIZATION

To assess whether engagement differed by hemisphere, we compared left and right aVPC in verbal WM retrieval and oddball detection – conditions in which both regions showed significant activity in the whole-brain analyses. *Post hoc t*-tests showed that the left hemisphere was more engaged in verbal WM retrieval than the right hemisphere [*t*(16) = 3.56, *p* = 0.003]. In contrast, there was no hemispheric difference in the extent of neural response in the oddball detection task [*t*(16) = 0.91, *p* = 0.37]. Note that this latter result argues against the idea that the aVPC simply reflects motor-related activity common to both retrieval and oddball detection. Both the verbal WM and oddball detection task required right-hand responses, but the left-hemisphere was not dominant in both tasks.

## DISCUSSION

In this study, we examined functional differences among subregions of the VPC by using two tasks that placed low or high demands on WM. VPC ROIs generated from the whole brain analysis of the oddball and WM task revealed different patterns of activation depending on the task. We found that more anterior regions of the VPC were active during the oddball task and WM retrieval, bilaterally, while posterior regions of the VPC showed dissociable responses; the left pVPC was engaged during the WM task, but not during the oddball task and the right pVPC showed the opposite pattern. In the following discussion we highlight the pattern of activity of the aVPC and pVPC in relation to that of previously published reports of VPC function.

### ANTERIOR VPC

We found regions of the left and right supramarginal gyrus that were sensitive to both oddball targets and WM retrieval. These regions, however, were not significantly active during the encoding or maintenance stages of WM. This pattern of results strongly suggests that the anterior VPC serves a non-mnemonic role in WM. Below we focus on potential contributions of the left aVPC to stimulus-driven attention, however, we note that other non-mnemonic functions such as context updating cannot be ruled out on the basis of our results.

Evidence for a ventral, frontoparietal pathway involved in stimulus-driven attention has accumulated over the past decade, and the right VPC is thought to be a critical node along this pathway (Corbetta et al., 2000; Todd et al., 2005; Serences and Yantis, 2007; Weissman and Prado, 2012; Chang et al., 2013; Chica et al., 2013). The right VPC is argued to trigger a shift of attention based on salient or task-relevant features of a stimulus. One possibility is that the left VPC provides a similar function to that of the VPC in the right hemisphere. We predicted that potential stimulus-driven attention functions of the VPC should be engaged whenever task-relevant stimuli are present, namely, at encoding and retrieval, but not during maintenance.

A region subserving stimulus-driven attention should be active at encoding given that attention prioritizes the access of information into WM (Gazzaley and Nobre, 2012) regardless of whether attention is directed voluntarily or by stimulus-driven factors (Cowan, 1999; Brown et al., 2000; Farrell and Lewandowsky, 2002). In fact, working memory performance is better for task-relevant items that capture stimulus-driven attention (Schmidt et al., 2002; Bays and Husain, 2008) especially at times when the ability to attend voluntarily is likely to be diminished (Ravizza and Hazeltine, 2013).

Stimulus-driven attention may also facilitate WM retrieval. First, stimulus-driven attention may be necessary for re-orienting attention to the retrieval probe. Stimulus-driven attention may be critical for shifting attention to the probe given that attentional resources are likely to be recruited for internal maintenance processes. Second, stimulus-driven attention may be captured by a salient aspect of the probe item. According to the attention to memory (AtoM) model, stimulus-driven attention is engaged when retrieval cues are salient; saliency in this context refers to either a highly novel item that has not been previously encoded (“new”) or that triggers a rich memory representation of an item that has been encoded (“old”; Kim and Cabeza, 2007; Cabeza et al., 2008). Consistent with a VPC role in stimulus-driven attention, the VPC was more engaged in high confidence “old” and “new” trials compared to low-confidence trials (Kim and Cabeza, 2007).

The results of our study provided only partial support for the stimulus-driven attention account of left aVPC function. In support of this account, the left aVPC was active at retrieval in response to the probe item. Moreover, activity of the anterior VPC was at baseline levels during maintenance when attention must be directed to the contents of memory rather than to new incoming information. Critically, greater activity during the maintenance interval predicted an error. This is consistent with other results showing that activity of the VPC is associated with errors when stimulus-driven attention is captured by irrelevant distractors (Olesen et al., 2007; Anticevic et al., 2009; Majerus et al., 2012). In fact, several studies have demonstrated that the more the VPC is suppressed during maintenance, the less likely salient visual information is to be detected or attended (Todd et al., 2005), and the more likely that the memory set will be recalled (Anticevic et al., 2009). The degree of suppression also seems to be load dependent as the VPC has been shown to be parametrically suppressed proportional with the number of items maintained in WM (Todd et al., 2005; Majerus et al., 2012).

Contrary to our predictions, there was little evidence that the aVPC was recruited at encoding. Although activity at encoding was above baseline, it did not survive whole-brain correction. It is possible that voluntary attention was sufficient for encoding items into WM. We have shown, for example, that stimulus-driven attention improves recall only when items are at the end of a list when voluntary attention was more likely to have lapsed (Ravizza and Hazeltine, 2013). Thus, this null result should be interpreted with caution, as stimulus-driven attention may not be necessary in our design for accurate encoding.

Thus, our findings are strongly supportive of a role of the aVPC in a non-mnemonic function rather than WM storage or phonological-motor integration. We found only partial support, however, for a specific role of the aVPC in stimulus-driven attention. Future studies are necessary to directly test whether the left VPC is necessary for triggering stimulus-driven attention to information at encoding and retrieval.

### POSTERIOR VPC

A left pVPC site spanning the STG and VPC was engaged at encoding and retrieval of the WM task, but not during the oddball task. *Post hoc* analyses revealed that activity in this region was sustained over the maintenance delay. This region is similar to the STG region observed in speech perception and production (Okada and Hickok, 2006; Acheson et al., 2011; Koenigs et al., 2011) and the region showing maintenance-related activity in verbal WM (Sakai et al., 2002; Hickok et al., 2003; Buchsbaum et al., 2005; Fiebach et al., 2006; Ravizza et al., 2011). Given its association with speech perception, this region is unlikely to serve as a dedicated WM buffer. Most likely, this region supports speech-related processing that supports both WM and perception, and is hypothesized to support verbal WM through its role in integrating phonological and motor codes (Hickok and Poeppel, 2000; Hickok, 2009). This finding supports the idea of WM storage as the reactivation of sensory processes or long-term memory representations rather than a buffer used only to maintain items for a short period of time (Ranganath et al., 2004; Buchsbaum and D’Esposito, 2008; Serences et al., 2009; Riggall and Postle, 2012).

In contrast, the pVPC in the right hemisphere was not reliably active in the verbal WM task, but was engaged in oddball detection. Given that both aVPC and pVPC in the right hemisphere were engaged in oddball detection, one possibility is that more cortical area is devoted to processes such as stimulus-driven attention or context updating in the right hemisphere compared to the left. In contrast, these non-linguistic processes may be restricted to more anterior parietal regions than those in the right hemisphere as a result of the lateralization of language functions on the left. It may be that more posterior regions of the VPC were recruited for language processing (Suchan and Karnath, 2011).

### COMMON AND DISSOCIABLE RESPONSES OF THE VPC

Functions of the left VPC appeared to be fractionated rather than unitary. The aVPC and pVPC showed different patterns of response in the WM and oddball tasks. The aVPC was active in oddball detection and WM retrieval which we have suggested is consistent with the stimulus-driven attention account. The pVPC did not show reliable activity in the oddball task, but was active during all stages of a WM task. Critically, the regions differed in their activity during maintenance; that is, maintenance engaged the pVPC whereas activity of the aVPC was at baseline. Moreover, aVPC activity in the maintenance stage was associated with making an error. Similar to Suchan and Karnath (2011), we suggest that the pVPC was adapted to process language and, additionally, that its role in speech perception also serves to maintain this information in WM. In contrast, anterior regions may have retained a similar function to its homolog in the right hemisphere, perhaps in stimulus-driven attention. Interestingly, the site we observed in oddball detection in the left aVPC is consistent with the more anterior site of lesions producing attentional impairments in atypical cases of left hemisphere neglect (Ogden, 1985). Thus, the aVPC might be important for WM because of its role in stimulus-driven attention.

In contrast, there was more support for a shared process in the right hemisphere. According to one account, this shared process is argued to be stimulus-driven attention (or bottom-up attention). Variations in the localization of VPC activity across tasks are explained by selective preferences for different types of information (Cabeza et al., 2012). In our explanation of VPC activity, we have capitalized upon this idea. Note, however, that the hypothesis that the VPC moves attention via stimulus-driven factors has been challenged (Carter and Huettel, 2013; DiQuattro et al., 2013). For example, responses of the right VPC to stimuli appear to be too late in the processing stream to provide a fast re-orienting signal (see Corbetta et al., 2008; DiQuattro et al., 2013 or a discussion of this issue). Instead, the right pVPC is argued to be a multi-modal association area important for creating and updating the sensorimotor context (DiQuattro et al., 2013) or decision-making context (Carter and Huettel, 2013). The present results cannot adjudicate between these alternative theories of VPC function. Instead, our findings are generally supportive of a potential unitary function of the right VPC.

### LIMITATIONS

The WM and attention tasks were presented in a fixed order with the WM task always preceding the oddball task. As explained previously, interleaving the tasks may have introduced a WM demand on the oddball task. Moreover, the WM task was more difficult than the oddball task and would be more vulnerable to fatigue if presented after the oddball task. It must be noted, however, that this fixed order may have affected the results. For example, participants may have expended less effort during the oddball task because it was at the end of the scan. It is also possible that the oddball stimuli were not very novel given that they had been previously presented in the WM task. Both of these factors may have diminished neural responses to oddball targets. Although oddball detection rate was very high, these potential effects cannot be ruled out and should be directly tested in future experiments.

Slow-event related designs are useful in isolating activity during multiple stages of a single event; however, this method has the disadvantage of reducing the number of trials per condition. We presented 20 trials per condition in the WM task which was further reduced due to (a small number of) errors. Signal values were potentially noisy which would reduce our power to observe the effects of our experimental manipulations. Future work could implement jittered fast-even related designs to balance the needs for more trials with the ability to isolate stages of interest.

## CONCLUSION

In this study, we observed dissociable patterns of activity in regions of the left and right VPC. The anterior VPC, bilaterally, was sensitive to novel targets in the oddball task and was active during retrieval of the WM task. We argue that the pattern of activity in this region is consistent with theories of the ventral parietal cortex as subserving a non-mnemonic process such as stimulus-driven attention. The data also suggest that the aVPC is unlikely to support verbal WM storage and, instead, maintenance is most likely to occur in regions involved in speech perception.

In contrast, posterior regions of the VPC/STG showed dissociable functions with the left hemisphere responding primarily in the WM task and the right hemisphere in the oddball detection task. This dissociation corresponds with the traditional way in which these areas have been characterized with verbal WM functions in the left hemisphere and attention functions in the right hemisphere.

## Conflict of Interest Statement

The authors declare that the research was conducted in the absence of any commercial or financial relationships that could be construed as a potential conflict of interest.
